# Lipoaspiration and Lymph Node Transfer for Treatment of Breast Cancer-related Lymphedema: A Systematic Review

**DOI:** 10.7759/cureus.6096

**Published:** 2019-11-08

**Authors:** Antonio J Forte, Maria T Huayllani, Daniel Boczar, Pedro Ciudad, Oscar Manrique

**Affiliations:** 1 Plastic Surgery, Mayo Clinic Florida - Robert D. and Patricia E. Kern Center for the Science of Health Care Delivery, Jacksonville, USA; 2 Plastic, Reconstructive and Burn Surgery, Arzobispo Loayza National Hospital, Lima, PER; 3 Plastic Surgery, Mayo Clinic, Rochester, USA

**Keywords:** breast cancer lymphedema, lymph node transfer, lipoaspiration, lymph node, lymphedema, microsurgery, microvascular free flap, surgical treatment, plastic surgery

## Abstract

Lipoaspiration and venous lymph node transfer have each been described as procedures that would improve symptoms of lymphedema. We aim to describe the efficacy of the combination of lipoaspiration and lymph node transfer and to report the outcomes in breast cancer-related lymphedema patients. The search was conducted by querying the PubMed, EMBASE, and Ovid Medline databases for studies that considered the use of lipoaspiration and venous lymph node transfer as surgical treatment for breast cancer-related lymphedema. Different combinations of the keywords “aspiration lipectomy” AND “lymphedema” AND “lymph node transfer” were used for the search. From a total of 20 articles, five met inclusion criteria. All patients included in these studies had stage II or III lymphedema. Two studies considered lipoaspiration as the first step followed by lymph node transfer, two considered lymph node transfer as the first step followed by lipoaspiration, and one applied both procedures simultaneously. A meaningful volume reduction was achieved in all cases. Patients who underwent lymph node transfer first followed by lipoaspiration appeared to have the best outcomes. This systematic review suggests that the combination of lymph node transfer and lipoaspiration is a potential surgical treatment that may improve outcomes achieved by one single procedure in patients with stage II to III breast cancer-related lymphedema.

## Introduction and background

Breast cancer-related lymphedema incidence varies from 9% to 41% in patients who undergo axillary lymph node dissection [1]. No cure for this condition exists; however, the current management of this disease is focused on decreasing symptoms and morbidity and improving the quality of life of these patients [2]. Surgical therapies are applied when conservative treatment has not resulted in effective outcomes. They include physiologic surgical techniques, such as the lymphovenous shunt procedures, lymphatic-lymphatic bypass procedures, and vascularized lymph node transfer, and ablative surgeries, such as liposuction and debulking surgery [3-4]. Other procedures like lymphovenous anastomosis or bypass and lymph node transfer techniques are suggested to be successful for early stages of lymphedema; however, they require more time and effort, are expensive, and have more potential adverse events after surgery, and their use as a single procedure has not been demonstrated to be suitable for late stages of lymphedema [5]. For chronic lymphedema treatment, liposuction was proposed to remove the hypertrophy of subcutaneous adipose tissue and favor the flow rate of lymph drainage [6-7]. Alternatively, lymph node transfer surgery consists of removing lymph nodes from a donor site and transferring as free flap to the affected limb. This last procedure is beneficial when no suitable lymphatics are available for lymphovenous anastomosis and it can be performed in continuity with the deep inferior epigastric artery perforator flap for breast reconstruction [8]. The lymph node transfer procedure is thought to enhance angiogenesis and lymphatic regeneration through the action of growth factors [9]. Moreover, the lymph nodes flaps are suggested to have a suction mechanism of lymphatic drainage in the implanted area.

Although lipoaspiration and lymph node transfer are usually performed as a unique surgical treatment without any agreement regarding timing, the use of both combined procedures to treat chronic lymphedema has been suggested to improve outcomes between these patients [1].

The purpose of this study was to review and summarize the studies to date that report outcomes regarding lipoaspiration in combination with lymph node transfer for the treatment of chronic breast cancer-related lymphedema. In addition, the surgical techniques used in the reported studies have been described.

## Review

Methods

Study Selection

This systematic review considered all studies reporting the use of lipoaspiration in combination with lymph node transfer in patients with breast cancer-related lymphedema. We followed the Preferred Reporting Items for Systematic Reviews and Meta-Analyses (PRISMA) guidelines to report our findings. Studies were included if they reported volume measurements of the affected limb of breast cancer-related lymphedema patients. Reviews and studies that did not specify outcomes for breast cancer-related lymphedema were excluded. All studies were written in English.

Data Sources and Search Strategy

One author (M.T.H.) conducted a systematic review on October 8, 2019, in the PubMed, EMBASE, and Ovid Medline databases, searching for articles reporting on lipoaspiration in combination with lymph node transfer for treatment of breast cancer-related lymphedema. The following terms were used for the search strategy: (((((((((((((((((Lipectomies[Title/Abstract]) OR Aspiration Lipectomy[Title/Abstract]) OR Aspiration Lipectomies[Title/Abstract]) OR Lipectomies, Aspiration[Title/Abstract]) OR Lipectomy, Aspiration[Title/Abstract]) OR Aspiration Lipolysis[Title/Abstract]) OR Lipolysis, Aspiration[Title/Abstract]) OR Suction Lipectomy[Title/Abstract]) OR Lipectomies, Suction[Title/Abstract]) OR Lipectomy, Suction[Title/Abstract]) OR Suction Lipectomies[Title/Abstract]) OR Lipolysis, Suction[Title/Abstract]) OR Suction Lipolysis[Title/Abstract]) OR Liposuction[Title/Abstract]) OR Liposuctions[Title/Abstract]) OR Lipoplasty[Title/Abstract]) OR Lipoplasties[Title/Abstract]) AND ((lymphedema[Title/Abstract]) OR lymphoedema[Title/Abstract]) AND (lymph node transfer). The included studies were uploaded into EndNote (Clarivate Analytics), and two independent reviewers (M.T.H. and D.B.) identified the final studies. Manuscripts were reviewed manually by one author (M.T.H.) and selected according to inclusion and exclusion criteria. First, studies were filtered based on the title and abstract. Then, for final selection, the full text of the selected studies was screened. When the reviewer was unsure whether to include a study in the final selection, another author (D.B.) reviewed the articles and, according to selection criteria, both reviewers came to a consensus for the final decision.

Data Pooling and Data Analysis

Extracted data were summarized and pooled. Author, year of publication, type of study, sequence of procedures, time between procedures, number of patients, age of patients, cause of lymphedema, stage of lymphedema, follow-up, and outcomes of included studies were presented.

Results

**Figure 1:**
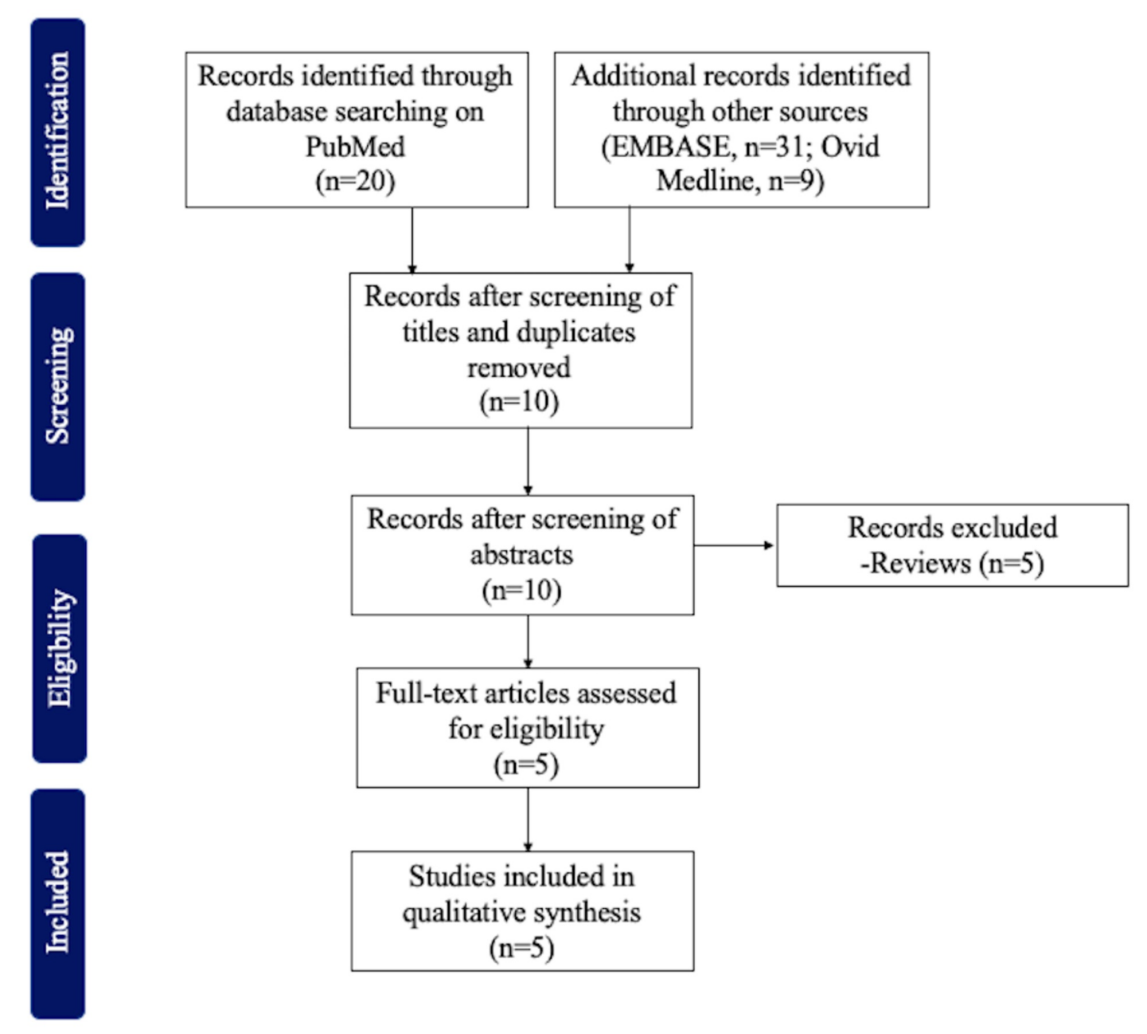
Inclusion and exclusion criteria

We found 20, 31, and nine articles in our PubMed, EMBASE, and Ovid Medline search, respectively. From these, only five articles met the inclusion criteria (Figure 1).

All included studies were published between 2014 and 2019. Study descriptions are provided in Table 1.

**Table 1:** Studies to date assessing outcomes of lipoaspiration and lymph node transfer combined procedure LN, lymph node transfer; LS, lipoaspiration [1], [10-13]

Author	Year	Type of Study	Sequence of Procedures	Time between procedures	Number of Patients	Age	Cause	Stage	Follow-up	Outcomes
Leppapuska IM et al.	2019	Prospective	Simultaneous	-	48 (LN+LS = 21, LN = 27)	Mean: LN+LS = 56.7, LN = 50.2	Breast cancer (n = 20), Hodgkin’s lymphoma (n = 1)	II	52 months	Volume reduction greater in LN+LS group.
Agko M et al.	2018	Prospective	1st: LN 2nd: LS	6 to 8 months	6	Mean: 52 (range: 27-72)	Breast cancer	II	23.5 months	Volume reduction, incidence of skin infections reduced
Cook KH et al.	2016	Case Report	1st: LS 2nd: LN	10 months	1	52	Breast cancer	III	12 months	Volume reduction
Nicoli F et al.	2015	Prospective	1st: LN 2nd: LS	1 to 3 months	10	Mean: 54.6+/-9.3	Breast cancer	-	-	Volume reduction
Granzow JW et al.	2014	Case Series	1st: LS 2nd: LN	11 months	2	55 and 63	Breast cancer	-	-	Volume reduction

The age of patients ranged from 27 to 72 years. Lipoaspiration or lymph node transfer was offered after conservative treatment in all studies. Lymphedema developed after breast cancer in almost all patients, except one whose lymphedema was secondary to Hodgkin's lymphoma [1,10-13]. Surgical procedures were applied in patients with stage II or III of lymphedema. The sequence between procedures varied in all studies; two considered lipoaspiration as the first procedure followed by lymph node transfer, two described lymph node transfer as the first step followed by lipoaspiration, and one reported performing both procedures at the same surgery [1,10-13]. The time between lipoaspiration and lymph node transfer when performed at different times ranged from 1 to 11 months. A meaningful volume reduction after both procedures was found in all studies.

Surgical Techniques

For lymphatic reconstruction, the lymph nodes can be harvested from the supraclavicular or groin area, along with the lymphatic vessels and fat [10-11]. Lymph node flaps from the supraclavicular area contain the transverse cervical artery in a sigmoid area of 1.5 cm and should be taken from the posterior triangle of the contralateral or ipsilateral side, with a skin island of 7 cm × 4 cm. If they are harvested from the groin area, an elliptical skin paddle of 6 cm × 4 cm should be harvested in parallel and inferior to the inguinal ligament and medial to the sartorius muscle and should include the superficial circumflex iliac artery and vein. This procedure can be combined with breast reconstruction using deep inferior epigastric perforator or muscle-sparing transverse rectus abdominis flap. In this case, the axillary scar should be removed and the thoracodorsal vessels prepared for the microvascular anastomoses depending on the lower abdominal flap. However, the best recipient site is usually the volar side of the wrist because the flexion of the wrist joint promotes lymph flow. The anastomosis should be done with the palmar branch of the radial artery and vein.

Liposuction can be performed before, after, or during the dissection of the lymphatic flap, following the dry technique, through the use of a tourniquet making compression of 250 mm Hg for a maximum of 60 minutes, or the wet technique, using 1 mg of epinephrine in 1,000 mL of 0.9% NaCl [10]. Finally, through microcannulas of 1 or 3 mm and small incisions, aspiration of the adipose tissue is achieved. In the postoperative care, patients should use compression level 2 garments for at least six months after the last surgery.

Discussion

This review critically evaluated the outcomes of combined lipoaspiration and lymph node transfer procedures as a treatment for breast cancer-related lymphedema. We found that all studies reported positive outcomes when both procedures were performed in combination, specifically for patients with stage II or III lymphedema, achieving an immediate decrease of volumes in the affected upper extremities.

Most of the studies evaluating the use of both procedures were observational, without a control group. Leppapuska et al. were the first to compare the outcomes of lymphedema patients treated with lymph node transfer and lipoaspiration with those of patients only receiving lymph node transfer [1]. They found a volume reduction of 87.7% in the group treated with both procedures, without any additional increase after a seven-day stop of use of compression garments in approximately 20 months of follow-up. Volume reduction was greater in the combined group, but the difference was not statistically significant (*P *= 0.28). The outcome of maintaining reduced volume seven days after removing compression garments following the combination therapy is interesting because compression garments are needed to maintain the reduced volume achieved in patients treated only with lipoaspiration, and removing compression garments for one week one year post liposuction has led to an increase in volume up to 370 mL [14-15]. Some studies have suggested that lymph node transfer promotes regeneration of lymphatic tissue three months after surgery [16-17]. This may be beneficial for patients as they can potentially stop using compression garments early and maintain the volume of the affected arm postsurgery.

Combination therapy was further found to achieve a decreased rate of skin infections compared to a group treated only with lipoaspiration (*P* = 0.02) [1]. This finding is supported by Agko et al. who also reported a decrease in the rate of skin infections [13]. Moreover, the circumference reduction rate of 37.8% reached after lymph node transfer in the upper extremity lymphedema increased to 97.8% after lipoaspiration [13]. In addition, lipoaspiration increases the blood flow to the skin, decreasing the rate of infections after removing the excess adipose tissue that predisposes to fibrosis due to the presence of the proinflammatory cells and cytokines, [18].

Regarding the question of which procedure should be performed first, there is still no consensus, and further studies are needed to compare both possibilities. It is thought that lipoaspiration could cause venous retention due to potential circulatory impairment when venous lymph transfer is performed first [12]. However, when lymph node transfer was performed before lipoaspiration, an approximately 90% improvement in the arm circumference reduction was achieved [11]. In addition, skin tonicity improved compared to the preoperative measurement. On the other hand, when lipoaspiration was performed first, there was an increase in volume in the affected upper extremity at one-year follow-up compared to the immediate postsurgical evaluation [12]. Hence, when patients undergo lipoaspiration first, there is a need to wait for six to 12 months after surgery to reach a steady-state volume to allow for venous lymph node transfer and obtain better outcomes. Moreover, significant volume reductions of 111% and a decrease in the incidence of cellulitis were found when lipoaspiration was performed first, followed by a lymph node transfer [10].

Other combined surgical procedures, such as venous lymph node transfer and lymphovenous bypass anastomosis, have previously been applied, both simultaneously and in sequence [19-20]. However, these procedures approached the lymphatic flow impairment, not the static component corresponding to the adipose tissue in chronic lymphedema. Lipoaspiration has also been assessed after lymphovenous anastomosis, with promising results, but the comparison of other combined procedures with lipoaspiration in combination with lymph node transfer surgery needs to be evaluated in further prospective studies [21]. 

Strengths and Limitations

In this systematic review, we described the studies assessing lipoaspiration combined with lymph node transfer procedures as the new surgical management for chronic breast cancer-related lymphedema. A limitation of this review is heterogeneity due to the nature of the studies, the presence of different protocols, and the follow-up of patients, which makes it difficult to compare results and perform statistical analysis. In addition, inherent limitations of the review methodology might be present, such as search, selection, and publication biases. However, we believe this systematic review is relevant as it describes the surgical technique, outcomes, and benefits of the use of lipoaspiration and lymph node transfer as a combined procedure for the treatment of breast cancer-related lymphedema.

## Conclusions

Lymph node transfer in combination with lipoaspiration is a potential surgical treatment for chronic lymphedema patients. Combining these procedures could reduce the rate of noncompliance for compression therapy, maintaining a reduced volume after surgical treatment for patients with stage II to III disease. Another advantage of this combination is the decreased incidence of skin infections. Further prospective studies comparing this combined procedure with single procedures should be conducted to determine the best surgical management of these patients.
